# Use of Dual-Modality Antibody Imaging for Assessment of Lymph Node Metastases in Head and Neck Cancer

**DOI:** 10.7150/thno.116640

**Published:** 2026-01-14

**Authors:** Nicole Meeks, Ashtyn G. McAdoo, Yu-Jin Lee, Ramsha Akhund, Gary Smith, Jared Grice, Joseph Hoang, Kurt R. Zinn, Marisa E. Hom, Michael C. Topf, Adam J. Rosenberg, Eben L. Rosenthal

**Affiliations:** 1 Department of Otolaryngology-Head and Neck Surgery, Vanderbilt University Medical Center, Nashville, TN, USA.; 2 Vanderbilt University Institute of Imaging Science, Vanderbilt University Medical Center, Nashville, TN, USA.; 3 Department of Otolaryngology-Head and Neck Surgery, Stanford University School of Medicine, Stanford, CA, USA.; 4 Department of Radiology and Radiological Sciences, Vanderbilt University Medical Center, Nashville, TN, USA.; 5 Department of Radiology, Michigan State University College of Human Medicine, East Lansing, MI, USA.

**Keywords:** head and neck cancer, radioguided surgery, fluorescence guided surgery, intraoperative detection, dual-modality

## Abstract

Surgical management of head and neck squamous cell carcinoma (HNSCC) patients often requires resection of the deep cervical lymph nodes. Fluorescence guided surgery (FGS) is a growing area of oncologic surgery that has shown promise for this purpose, however, it is limited by the signal penetration through the neck. To address this, we sought to evaluate the efficacy of the anti-EGFR antibody [^111^In]panitumumab to detect metastatic lymph nodes both on preoperative imaging and intraoperatively. We hypothesized that the addition of a radiolabeled antibody would be safe and effective in detecting malignant tissue when given alone or together with the optical agent panitumumab-IRDye800CW (pan800).

**Methods:** Seventeen patients were enrolled and received 5 mCi of [^111^In]panitumumab with nine patients (53%) receiving [^111^In]panitumumab and pan800. SPECT/CT scans were performed prior to surgical resection. Intraoperatively, patients underwent gamma tracing and the patients who received pan800 also underwent optical fluorescence imaging. Resected specimens were compared to final histopathology to determine the sensitivity and specificity of the two tracers.

**Results:** No adverse events related to the [^111^In]panitumumab were reported. SPECT/CT performed 3 days post-injection showed higher tumor-to-blood pool ratio compared to earlier scans (p = 0.04), and when compared to pathological assessment, could detect disease greater than 1 cm in diameter. Intraoperatively, resection of the primary tumor resulted in a significant drop in gamma counts (p < 0.001). *In vivo* detection of metastatic lymph nodes using the radiotracer was inconsistent and attributed to high background counts as patients who underwent surgery before 48 hours post-injection had significantly higher precordial counts than patients who underwent surgery after 48 hours, even when corrected for time, weight, and dose (p = 0.04). *Ex vivo*, metastatic lymph nodes had gamma counts almost twice that of benign (p < 0.0001). The separately dosed pan800 and [^111^In]panitumumab showed strong co-localization within metastatic lymph nodes (R = 0.87).

**Conclusion:** Intravenous infusion of [^111^In]panitumumab in HNSCC patients is safe, whether administered alone or with pan800. Consistent with other studies, *ex vivo* application of the radiotracer differentiated tumor containing tissue from benign. However, intraoperative use of the radiotracer to detect metastatic lymph nodes was limited by background signal at early timepoints and signal decay at later timepoints.

## Introduction

Presence of lymph node metastases is a negative prognostic indicator in solid tumors and often surgical removal for pathological assessment is required to properly stage patients. For example, in head and neck squamous cell carcinoma (HNSCC), current imaging modalities will miss 30-50% of occult metastases 1, 2. Lymph node removal is therefore necessary in clinically and radiographically nodal-negative patients, and an elective neck dissection is used to remove the cervical lymph nodes for diagnostic and therapeutic benefit. Elective neck dissection has shown to decrease nodal recurrence, while increasing disease-free and overall survival, but a large portion of patients will not have metastases detected on final pathology 3-5. Therefore, novel methods for focused preoperative or intraoperative identification of lymph node metastases are required.

Minimally invasive surgical techniques such as sentinel lymph node biopsy (SLNB) are associated with less morbidity, however, beyond the oral cavity, lack widespread adoption in HNSCC due to the region's complex anatomy and difficult access 6. Fluorescence-guided surgery (FGS) is another method of targeted intraoperative tumor identification that is gaining popularity in oncologic surgery 7. Although several tumor-specific optical agents are currently in clinical trials, many have adopted optically labeled monoclonal antibodies 8. Using this technology, cancer can be visualized intraoperatively by the surgeon in real time. Panitumumab-IRDye800CW (pan800) is a fluorescently labeled anti-epidermal growth factor receptor (EGFR) antibody that has been studied in HNSCC, where 90% of the tumors overexpress EGFR, as well as pancreatic and brain cancer 9-12. Pan800 has been shown to be safe, provide good tumor-to-background contrast for margin assessment, and demonstrate high specificity for cancer 13, 14. Additionally, previous trials have demonstrated the molecular imaging agent's ability to detect metastatic lymph nodes intraoperatively and during pathologic processing, aiding both the surgeon and pathologists 15-17. One important limitation of near-infrared dyes like IRDye800 is the shallow signal penetration (3-5 mm) 18. This means the lymph node basin must be widely exposed with a large incision to detect the signal in underlying tissue, and even after full exposure, deep-seated metastases may be missed due to signal obstruction.

Radiotracers are another possible method for targeted evaluation. Gamma-emitting radionuclides such as indium-111 penetrate deeper through tissue, are detectable on SPECT/CT allowing for cancer-specific preoperative imaging, and can be located intraoperatively using existing surgical equipment. Clinical investigation of radiolabeled monoclonal antibodies for intraoperative detection of colorectal cancer began over 20 years ago with success at that time 19-22. Since then, the utility of radioguided surgery (RGS) has been explored beyond monoclonal antibodies in several solid organ malignancies but has not been developed as a single agent for commercial application 23-26. Additionally, the concept of dual-labeling imaging agents with a radionuclide and fluorescent dye has been demonstrated in many pre-clinical studies and hypothesized to be beneficial 27-31. While clinical trials investigating dual-labeled antibodies are limited, the culmination of data have found radiolabeled monoclonal antibodies to be safe and helpful when used alone and in conjunction with IRDye800 32-36. In head and neck cancer specifically, one publication has explored the use of [^89^Zr]panitumumab for preoperative PET/CT given with pan800 for intraoperative fluorescence guided surgery. Along with demonstrating the radiotracer and fluorescent antibody were safe to give together, the results showed a strong correlation of the radiotracer SUV and fluorescence within metastatic lesions 37. However, [^89^Zr]panitumumab is limited for intraoperative detection using commonly available surgical equipment based on its emission pattern. While no studies report on the use of [^111^In]panitumumab in humans, pre-clinical studies in mice have shown the agent to have similar biodistribution to [^89^Zr]panitumumab and have no hematologic, renal, or hepatotoxicity 38, 39.

No previous publications have investigated the use of radiolabeled monoclonal antibodies for intraoperative detection in HNSCC alone or when paired with optical imaging. More importantly, targeted dual-modality antibodies have not been assessed for the identification of tumor-positive lymph nodes in any tumor type. Therefore, the goal of this study was to assess the feasibility of [^111^In]panitumumab alone and in conjunction with pan800 for preoperative and intraoperative detection of lymph node metastases in patients with HNSCC.

## Methods

### Clinical trial design

Two prospective, non-randomized, pilot clinical trials evaluating the use of [^111^In]panitumumab alone (NCT05901545) and with pan800 (NCT05945875) were approved by the Vanderbilt University Medical Center (VUMC) institutional review board. The Food and Drug Administration (FDA) approved [^111^In]panitumumab (IND 166143) and pan800 (IND 119474) as investigational drugs. Both studies were performed in accordance with the Declaration of Helsinki, ICH-GCP guidelines, and the United States Common Rule.

Eligible patients included adults with primary or recurrent HNSCC undergoing standard-of-care surgical resection and neck dissection. From 07/2023 and 01/2025, a total of 17 patients were enrolled between the two studies. All patients provided written informed consent. In the [^111^In]panitumumab alone (In-pan alone) cohort, patients received an intravenous loading dose of 100 mg unlabeled panitumumab, followed by a bolus injection of 4-6 mCi [^111^In]panitumumab. In the [^111^In]panitumumab and pan800 (In-pan+pan800) cohort, a flat dose of 50 mg pan800 was administered intravenously followed by a 4-6 mCi injection of [^111^In]panitumumab. The unlabeled loading dose and pan800 dose were determined by prior studies from another institution (unpublished) as well as prior experience with pan800 in HNSCC 14, 40, 41. All infusions happened 1-7 days prior to surgery and patients were monitored for 30 minutes following infusion of the study drugs. EKGs were obtained prior to injection and after injection to monitor for clinically significant QTc prolongation. Between infusion day and surgery, all patients underwent a SPECT/CT scan. Up to 7 days after antibody infusion, patients underwent standard-of-care surgery and accumulation of the study drug was assessed intraoperatively. Adverse events were assessed for 15 days following infusion and graded based on the National Cancer Institute Common Terminology Criteria for Adverse Events (CTCAE v5.0) 42. Study-related adverse events were defined as those deemed possibly related, probably related, or definitely related to the study drugs. Safety was determined by the absence of events related to the [^111^In]panitumumab or pan800 as well as absence of radiologic toxicities.

### Study drug preparation

All study drugs were produced under current Good Manufacturing Practice (cGMP) conditions. Production of pan800 has been described previously 40, 43. [^111^In]panitumumab was produced by the Vanderbilt University Institute of Imaging Science (VUIIS) Radiochemistry Core under IND 166142. In brief, 70 mg of Panitumumab was incubated with 3 equivalents of S-2-(4-Isothiocyanatobenzyl)-diethylenetriamine pentaacetic acid (SCN-Bn-DTPA) for 2 hours at 37ºC. The conjugate was then purified on a high-performance liquid chromatography (HPLC) size exclusion column using 0.9% saline as the mobile phase. The ratio of DTPA to Panitumumab was determined via Matrix-assisted laser desorption ionization time-of-flight mass spectrometry (MALDI-TOF MS). Concentration, purity, and other quality attributes of the conjugate were determined by HPLC. In a sterile plastic reaction vessel, the [^111^In]Indium (III) chloride solution in 0.05 HCl was neutralized by sodium acetate buffer to pH 4. The In-111 solution was then mixed with 1.0 mg of conjugated protein in a mixture of 0.9% saline and sodium acetate buffer. The reaction was incubated for 30 minutes at room temperature. The crude product was purified on a size-exclusion HPLC column using 0.9% saline as the mobile phase. The purified product was diluted to 6 mL with 0.9% saline and transferred to a vented sterile vial through a sterilizing filter. Samples were then removed for analysis of product quality. Radiochemical purity as determined by HPLC: 99.45 ± 0.79%; Specific activity: 40.71 ± 12.81 mCi/mg.

### SPECT/CT acquisition

After study drug infusion and prior to surgery, all participants underwent imaging of the head and neck with a dual head SPECT/CT system (NM/CT 670 or NM/CT 870c, GE HealthCare, Milwaukee, WI). A low-dose, non-contrast enhanced CT scan was obtained for attenuation correction and image co-registration. SPECT images were obtained using a medium-energy general purpose (MEGP) collimator for the NM/CT 670 system or a wide-energy high-resolution sensitivity (WEHRS) collimator for the NM/CT 870c system. Acquisition parameters included 64x64 matrix and 60 stops at 40 sec/stop. Two 20% energy windows centered on 171 keV (154-188 keV) and 245 keV (221-270 keV) were used. Images were reconstructed using ordered subset expectation maximization (OSEM) with 10 subsets and 2 iterations, incorporating attenuation correction and resolution recovery. In-111 scatter correction was applied when available (NM670). Presence and uptake of [^111^In]panitumumab within the primary tumor, regional lymph nodes, and blood was quantified by a nuclear medicine physician using MIM software (MIM version 7.2.7, MIM Software Inc., Cleveland, OH). Maximum count density in the tumors and lymph nodes were measured. To quantify blood pool, a standard size sphere was used to obtain maximum count density of both internal jugular veins and averaged by individual patient.

### Intraoperative antibody detection

On day 1-7 following study drug infusion, patients underwent standard-of-care surgery determined by their clinical care team. In all patients, localization of [^111^In]panitumumab was performed *in vivo* by obtaining counts per 10 seconds (10s) using a commercially available gamma probe (Navigator 2.0, Dilon Technologies Inc., Newport News, VA). For all counts, surgeons were instructed to hold the probe aimed at a 90-degree angle towards the floor and make direct contact with the tissue being measured to avoid detecting counts within confounding regions such as the liver or primary tumor when measuring the neck. *In vivo* background counts were taken from the liver, arm, leg, and precordial region where precordial counts were used to estimate circulating radiotracer as previously described 21. Back table 10s gamma counts were obtained on the primary and neck specimens *ex vivo*. After obtaining counts, the neck specimens were assessed by level as the fibrofatty specimens containing lymph nodes (packet) and any palpated lymph nodes were separated for individual 10s gamma counts. Individual lymph nodes were marked and sent to pathology routine with the remainder of the specimens. Any individual lymph nodes that could not be correlated to intraoperative back table counts were discarded from analysis.

In addition to gamma counting, *in vivo* fluorescence imaging was performed in the In-pan+pan800 patients on the primary and neck dissection site pre- and post-resection using an open-field, near-infrared imaging device (pde-neo II & PDE-GEN3, Hamamatsu Photonics K.K., Japan). *Ex vivo* closed-field fluorescence imaging of pan800 was performed on all resected specimens using a modified PEARL Trilogy device (LI-COR Biosciences Inc.).

### Tissue analysis

All specimens underwent routine standard-of-care processing. After formalin-fixation, tracked lymph nodes were placed in individual cassettes. Specimens from patients who received pan800 were imaged using the closed-field PEARL Trilogy (LI-COR Biosciences Inc.) and then grossed as usual. Specimens were re-imaged using the closed-field device after all specimens were placed in cassettes. After paraffin embedding, final pathology on haematoxylin and eosin (H&E) stained sections was determined by a board-certified pathologist. Quantitative assessment of the closed-field images was performed by drawing a manual region of interest (ROI) around lymph nodes to calculate the mean fluorescence intensity (MFI) using ImageStudio (LI-COR Biosciences Inc.), as described previously 15, 17. Additional regions of interest were drawn around patient-matched sections of fibroadipose tissue to calculate lymph node signal-to-background (SBR) using the fatty tissue MFI as background.

### Statistics

Data analysis, descriptive statistics, and figures were conducted using R Studio. For SPECT/CT scans, tumor-to-blood pool (TBP) ratio was calculated by taking the maximum count density of the primary tumor and dividing by the average maximum count density obtained from the jugular veins. All gamma counts were decay corrected for time of infusion using the formula: *Corrected Count = Measured Count x e^(0.693 x elapsed time)/67.2^* where elapsed time was measured in hours from infusion and 67.2 is the half-life of indium-111. Corrected counts were then converted to normalized counts per second (CPS_norm_) by correcting for kilograms of lean body mass (LBM) and injected dose using a method described previously by Mix et al. 23 with the equation: 
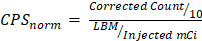
. Because the submandibular glands are known to express EGFR, level 1b packets were excluded from the *ex vivo* analysis of the radiotracer.

Data is presented as mean ± standard deviation (SD). For normally distributed data such as QTc intervals, a paired or unpaired t-test was used. Non-normally distributed data such as gamma counts and MFI were assessed using a paired or unpaired Wilcox-Rank Sum test. Optimal thresholds were calculated using Youden's index based on receiver operating curves (ROC) and used to determine sensitivity, specificity, positive predictive value, negative predictive value, and accuracy. Correlation between MFI and gamma counts are represented as Spearman's correlation coefficient. Boxplot representations follow standard format with the median as center line, first and third interquartile range as hinges, and whiskers representing 1.5*IQR from the hinge. For all confidence testing, a p-value of 0.05 or less was considered statistically significant and represented graphically as: *p < 0.05, **p < 0.01, ***p < 0.001, ****p < 0.0001, or NS not significant.

## Results

### Patient characteristics and safety

**Figure 1:**
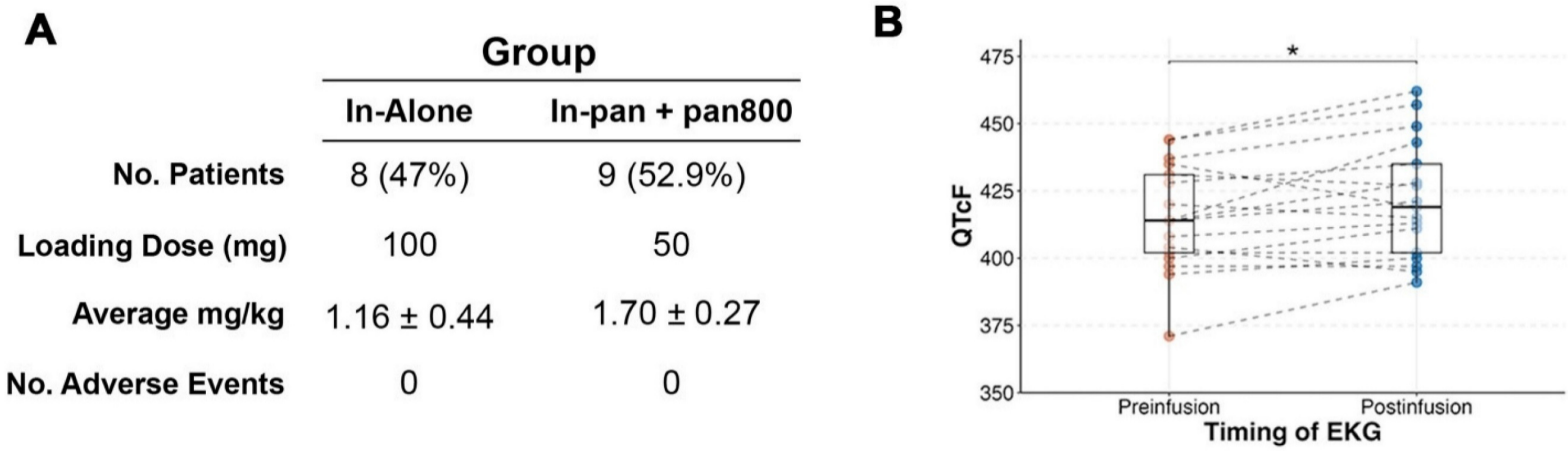
[^111^In]panitumumab is safe when used alone or when paired with panitumumab-IRDye800. **(A)** Study breakdown including average milligram per kilogram (mg/kg) between patients. Adverse events are those defined as being deemed possibly, likely, or definitely related to the study drug and graded by CTCAE v5.0. **(B)** Pre-infusion and post-infusion QTc intervals.

A total of 17 patients received [^111^In]panitumumab prior to undergoing surgery. The majority were male (76%), and the mean age overall was 63 ± 11 years. Eleven patients (65%) were clinically staged as nodal positive and seven patients (41%) had metastasis on final pathology. Patient characteristics including subsite, recurrence status, and time from infusion to imaging and surgery can be found in Table 1. The QTc interval increased post-infusion from 415 ± 20 to 421 ± 22 after infusion with an average percent change of 1.5 ± 2.7% (Figure 1). As we have reported previously 13, the difference between pre-infusion and post-infusion QTc was statistically significant (p = 0.03), but not clinically significant. No adverse events occurred that were possibly, probably, or definitely related to the study.

### SPECT/CT

**Figure 2:**
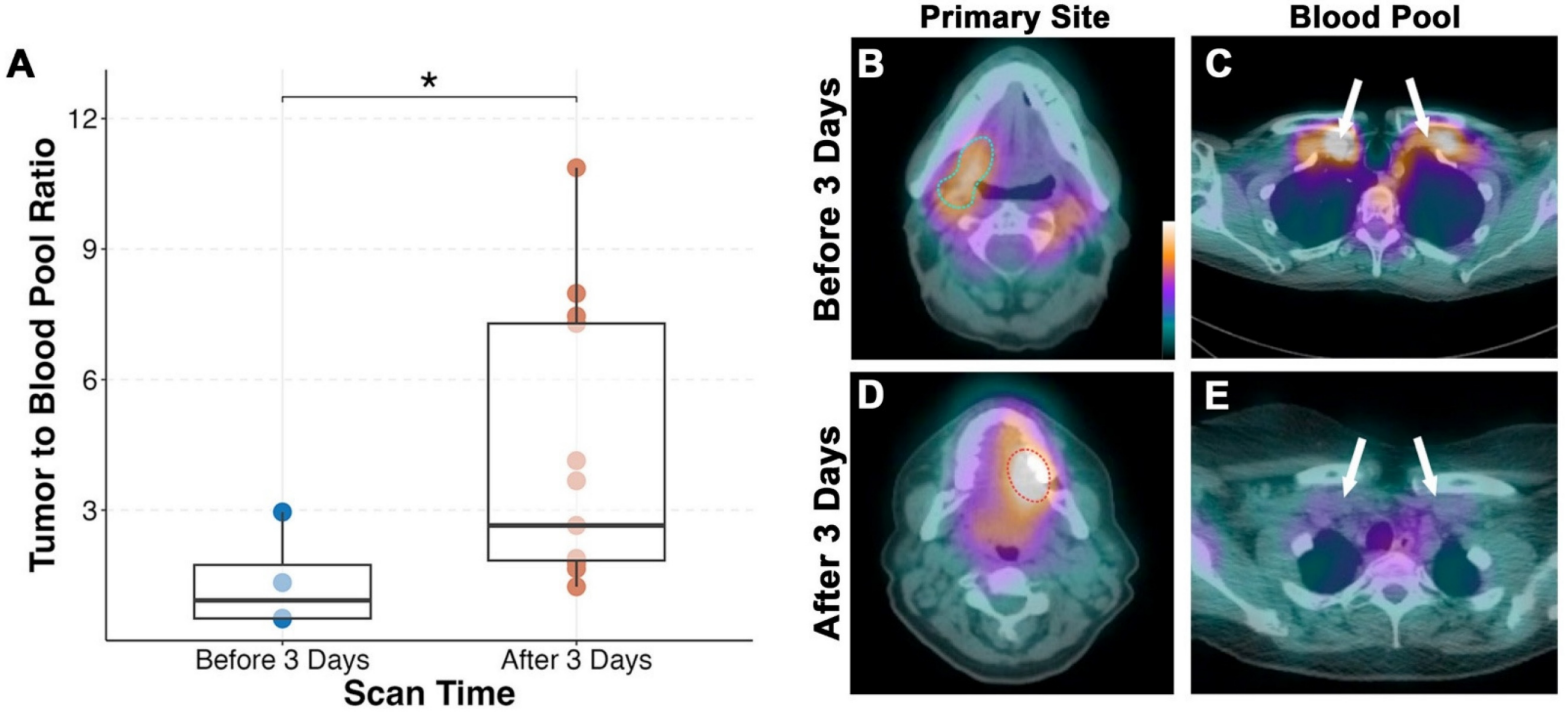
Performing SPECT/CT imaging of [^111^In]panitumumab after day 3 yields a better tumor-to-background ratio **(A).** Representative scans demonstrating mild tumor activity (outlined) in a patient scanned before day 3 **(B)** with higher subclavian blood pool as shown by the white arrows **(C)**. Comparatively, higher primary tumor activity **(D)** and less subclavian blood pool **(E)** is seen in a patient scanned after day 3.

Average time between infusion and SPECT/CT imaging was 3.7 ± 1.6 days. We found that patients imaged before 3 days (n = 4) had significantly lower tumor-to-blood pool ratio compared to those scanned after 3 days (p = 0.04; Figure 2). Fifteen of the seventeen patients (88%) had their primary tumor visualized on SPECT/CT. Both patients without detectable primary tumors on SPECT/CT imaging had tumors less than 1 cm in diameter on final pathology. The smallest detectable tumor was 1.5 cm in diameter and the mean detected tumor size was 3.7 ± 1.8 cm.

**Figure 3:**
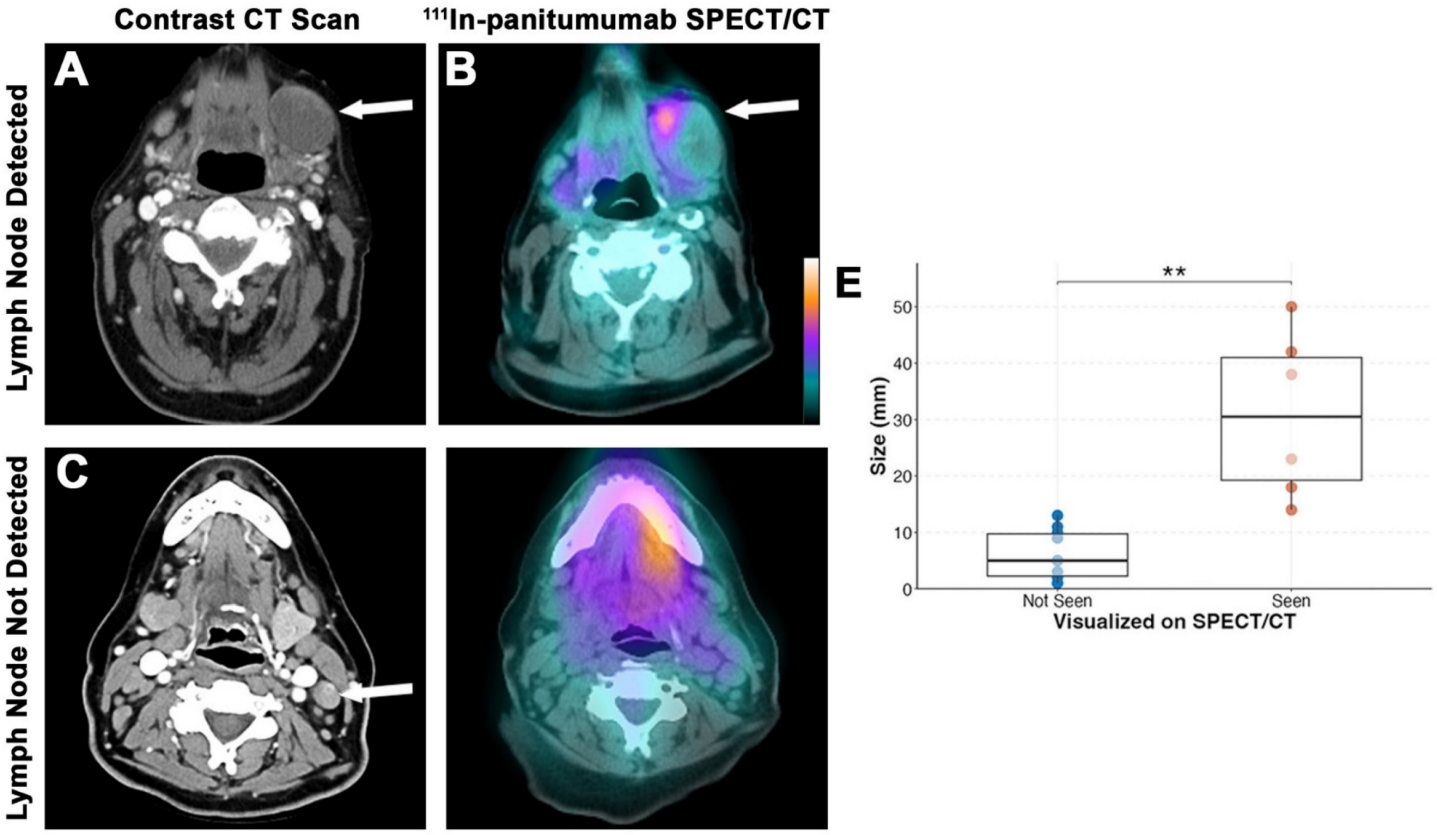
SPECT/CT detection of lymph nodes. Representative preoperative contrast-enhanced CT scan **(A)** and [111In]panitumumab scan **(B)** where a large metastatic lymph node measuring 5 cm in diameter on final pathology had clear localization of the radiotracer. Representative preoperative CT scan **(C)** from a different patient showing a smaller suspicious lymph node with calcification that was not definitively localized on [111In]panitumumab SPECT/CT **(D)**. Size breakdown **(E)** of positive lymph nodes seen compared to neck location **(F)**.

A total of 647 lymph nodes were surgically removed during neck dissection, of which 631 (97.5%) had no evidence of disease. Out of the 16 metastatic lymph nodes, six (37.5%) were detected on SPECT/CT (Figure 3). Consistent with our primary tumor findings, lymph nodes with metastasis smaller than 1 cm were not visualized on SPECT/CT imaging. The average size of detected lymph nodes and non-detected lymph nodes were 3.1 ± 1.5 cm and 0.6 ± 0.4 cm, respectively (p = 0.001). The sensitivity of SPECT/CT for detecting lesions was 63.6% and the negative predictive value (NPV) for all lesions combined was 98.1%.

### Intraoperative radiotracer detection

**Figure 4:**
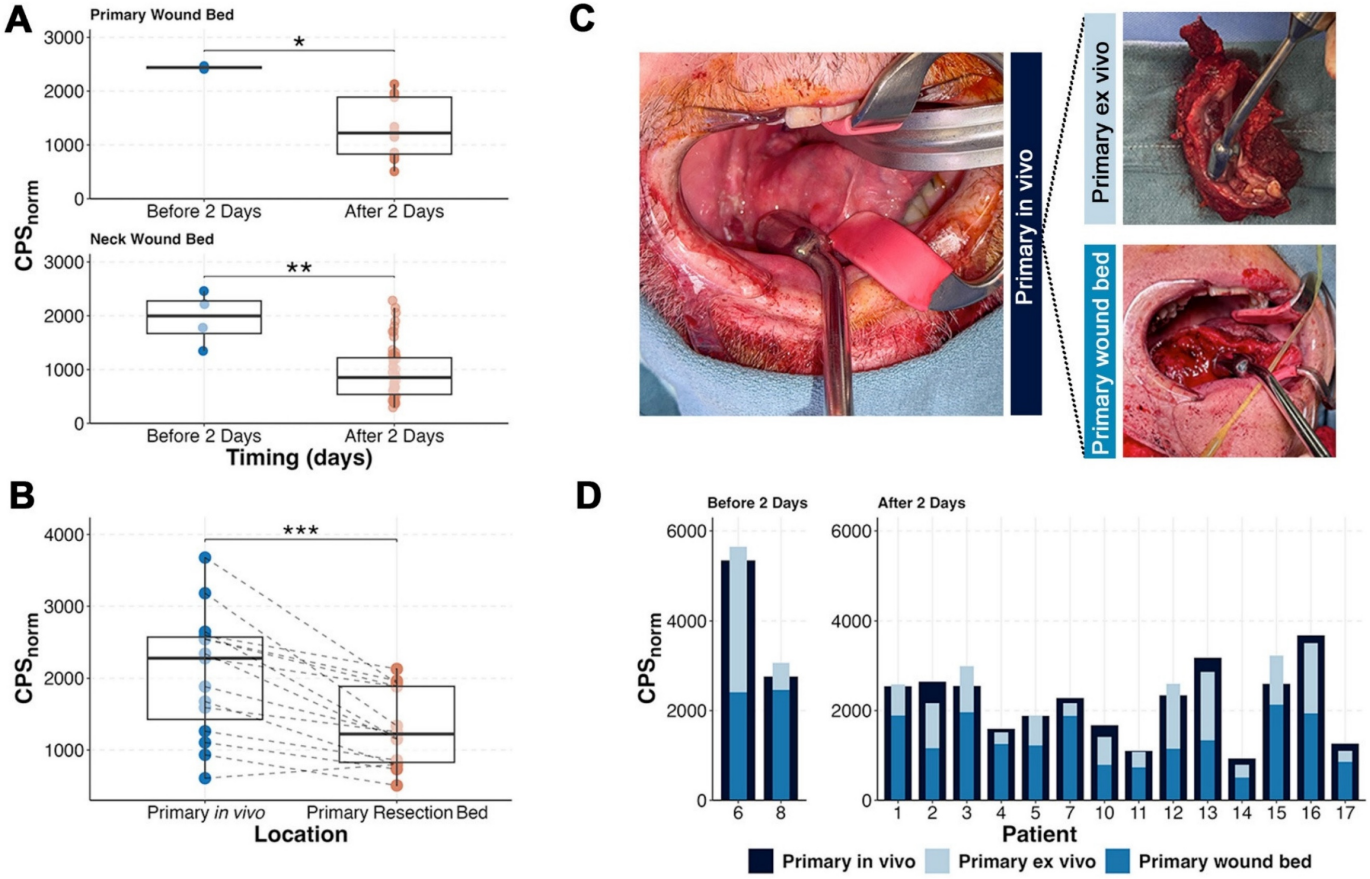
Timing and tracer localization in the primary tumor. **(A)** After tissue resection, patients who underwent surgery within 2 days of infusion had higher normalized primary and neck wound bed counts indicating high activity of remaining circulating tracer. **(B)** In patients with surgery performed after 2 days, removal of the primary resulted in a significant reduction of gamma counts. **(C)** Representative intraoperative images showing how the primary before resection (left), *ex vivo* (top right), and wound bed (bottom right) were measured. **(D)** Although counts varied amongst patients, the wound bed and the removed specimen result in a sum that is within range of the primary specimen before resection.

The average time from infusion to surgery was 4.75 ± 1.4 days. Surgery prior to 2 days was limited by non-specific detection of circulating radiotracer as demonstrated by the significantly higher normalized counts per second (CPS_norm_) within the precordium (p = 0.04), post-resection wound bed (p = 0.01) and post-lymphadenectomy wound bed (p = 0.003; Figure 4). Evaluation of patients who underwent surgery in excess of 2 days post-infusion showed that the primary tumor had significantly higher counts (2059 ± 863) compared to the wound bed after removal of the tumor (1308 ± 530; p < 0.001). Not unexpectedly, summation of *ex vivo* primary tumor counts with the wound bed counts approximated the *in situ* primary tumor (Figure 4C-D) suggesting [^111^In]panitumumab localization to the tumor.

**Figure 5:**
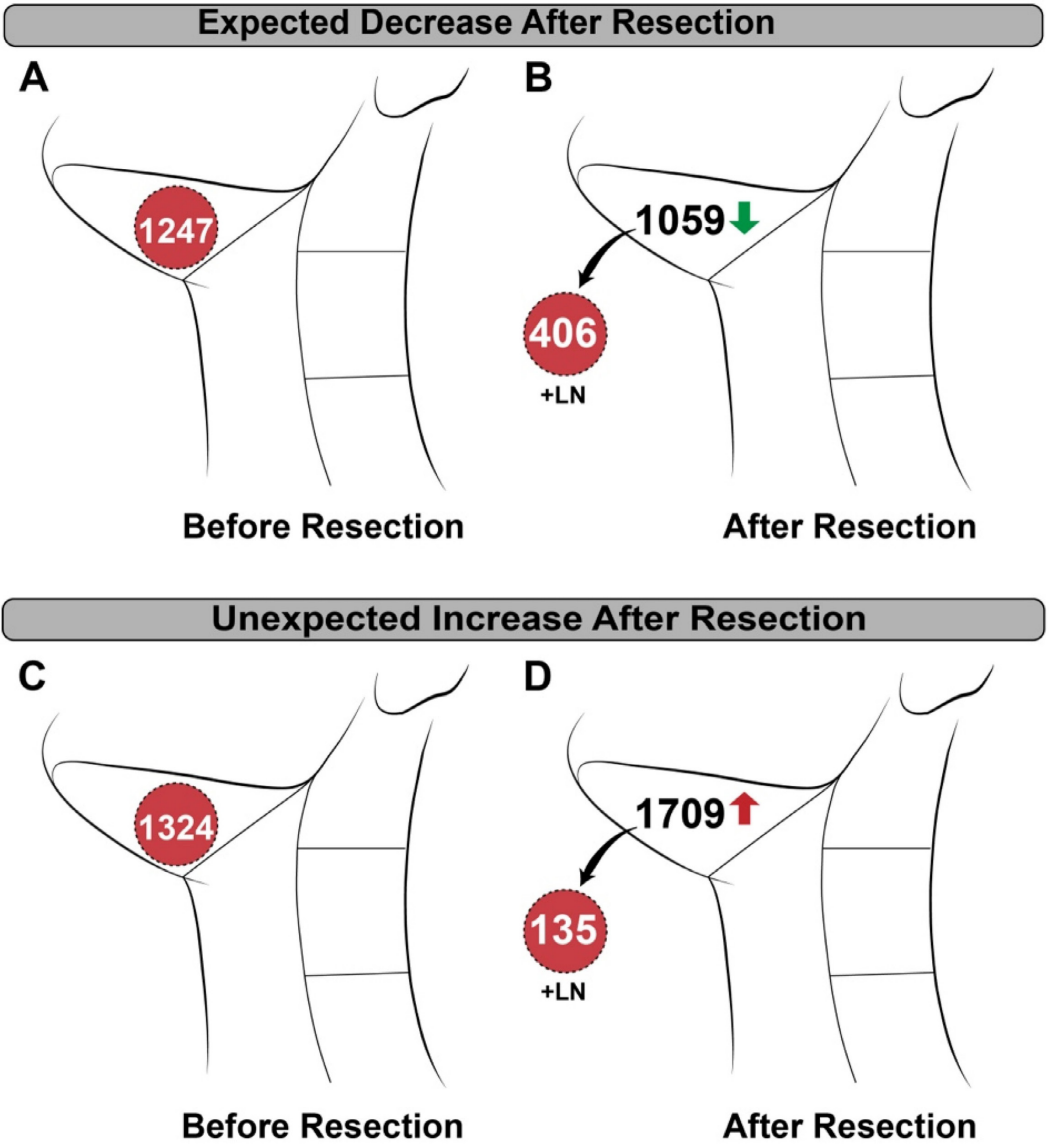
Patient examples of *in vivo* neck counting demonstrating variability. **(A)** Pre-resection and **(B)** post-resection normalized counts per second (CPS_norm_) in a patient with known metastasis showing an expected decrease in counts after removal. **(C)** Pre-resection and **(D)** post-resection CPS_norm_ of a different patient with known metastasis showing an unexpected increase after removal.

*In vivo* detection of lymph nodes was limited by detection of high background in the jugulodigastric chain and there was unexpected variability between patients. As shown in Figure 5, removal of the metastatic tissue resulted in a decrease of counts from pre- to post-resection in some patients, while removal in others resulted in an unpredicted increase in counts. Unlike the primary tumor, addition of CPS_norm_ from *ex vivo* lymph nodes and the post-neck dissection wound bed did not add up to the pre-resection CPS_norm_ values.

**Figure 6:**
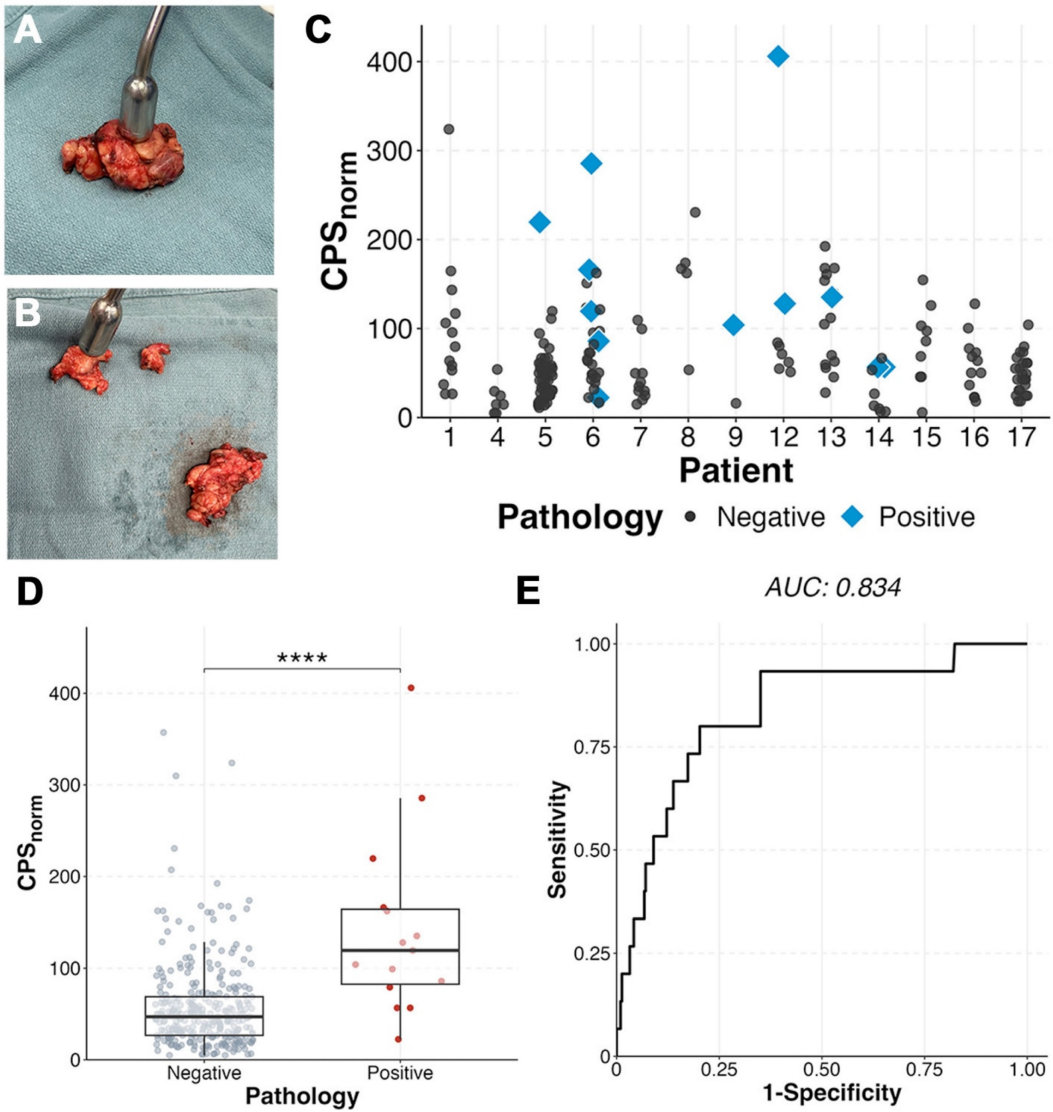
*Ex vivo* gamma counting demonstrates ability to detect metastases. **(A)** Example of fibrofatty tissue packets containing lymph nodes and then further dissected out to individual lymph nodes **(B)** and measured. **(C)** *Ex vivo* normalized counts per second (CPS_norm_) of patients who had multiple dissected lymph nodes able to be correlated to histopathology. Positive tissue (**D**) demonstrated higher CPS_norm_ than benign (p < 0.0001). **(E)** Receiver operative curve (ROC) of benign vs malignant tissue showing an optimal cutoff of 79, yielding a sensitivity of 80.0%, specificity of 79.4%, positive predictive value of 16%, and negative predictive value of 98.8%.

Assessment of *ex vivo* neck levels as fibrofatty tissue packets containing lymph nodes or individual lymph nodes showed the radiotracer identified positive lymph nodes when compared to benign nodes within the same patient. Metastatic lymph nodes (n = 15) had a two-fold higher CPS_norm_ (142 ± 99; Figure 6) than benign (57 ± 48; p < 0.0001). A metastatic lymph node had the highest gamma count in 5 of the patients (71%) with metastases on final pathology. In six patients (85.7%) three lymph nodes with the highest CPS_norm_ scores could have determined the patient's nodal status (metastases present vs absent) (Figure 6C). Area under the curve (AUC) was 0.834 with an optimal CPS_norm_ of 79 giving a sensitivity of 80%, specificity of 79.7%, and accuracy of 79.7%.

### Lymph node dual-modality

**Figure 7:**
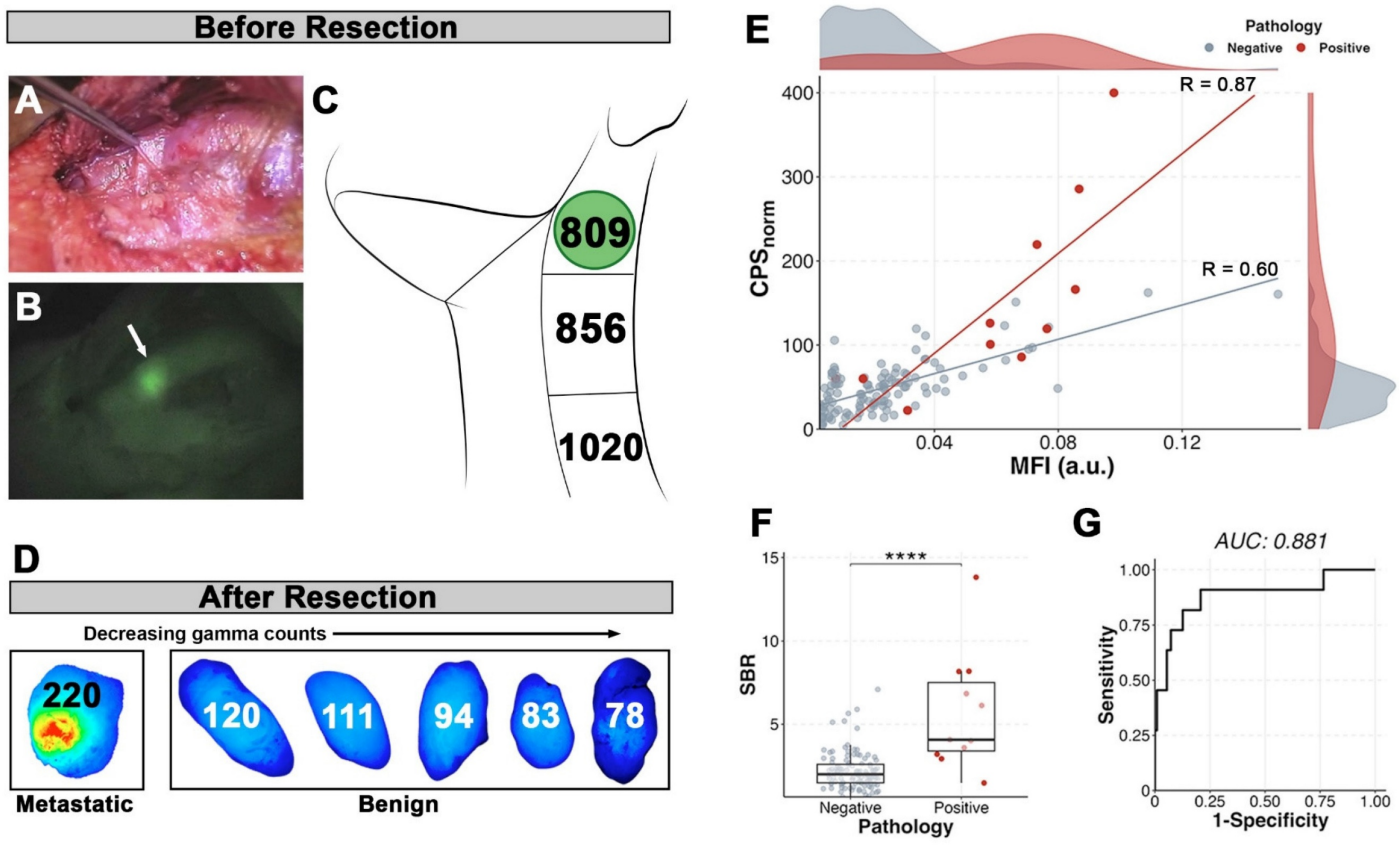
Fluorescence compared to the radiotracer. Patient example of visualized signal-to-background contrast within a metastatic lymph node *in vivo* prior to resection in brightfield **(A)** and fluorescence** (B)** with the metastatic lymph node denoted by the white arrow. In contrast, the *in vivo* radiotracer counts **(C)** were unreliable in indicating presence of the metastatic lymph node (indicated by green circle). **(D)** The top six lymph nodes determined by *ex vivo* gamma counts (numbers within) arranged by decreasing values and shown with their corresponding closed-field fluorescence imaging. **(E)** Correlation of *ex vivo* gamma counts and closed-field fluorescence of fresh lymph nodes from all patients shows strong correlation within metastatic lymph nodes (red line; R=0.87, p < 0.001). **(F)** Metastatic lymph nodes have higher signal-to-background (SBR) than benign (p < 0.0001). **(G)** Receiver operating curve (ROC) of SBR using benign fatty tissue as background yields a cutoff of 2.88 and sensitivity of 90.1%, specificity of 79.3%, positive predictive value of 30.0%, and negative predictive value of 98.9%.

There were a total of nine patients who received both [^111^In]panitumumab and pan800. Five of the six patients (83.3%) with metastases on final pathology had *in* vivo visualization of pan800 within positive lymph nodes. A representative patient comparison of the fluorescence and gamma counting can be seen in Figure 7, which demonstrates background activity was not a limiting factor *in vivo* for optical imaging but was a significant problem for the radiotracer. Interestingly, there was one patient with prior radiation who did not have a positive lymph node visualized on near-infrared fluorescence (NIRF) imaging, however, the metastatic tissue had noticeably higher CPS_norm_ compared to other resected tissue in this patient (286 vs 166 for the next highest count).

When dissected free of the fibrofatty tissue for pathologic assessment, there were 122 lymph nodes for which nuclear and optical data were available. Similar to the radiotracer, pan800 showed significantly higher SBR within metastatic lymph nodes than benign (p < 0.0001). CPS_norm_ and closed-field fluorescence were strongly correlated in metastatic tissue (n = 11; R = 0.87), demonstrating co-localization of the separately labeled antibodies (Figure 7E). Four patients had multiple lymph nodes with consistent data using both modalities. Two patients had a metastatic lymph node with both the highest CPS_norm_ and the highest MFI value. In the other two patients, a metastatic lymph node had either the highest CPS_norm_ value or highest MFI value, suggesting the utility of both imaging modalities. The AUC for SBR was 0.882 with an optimal cut-off of 2.88 yielding a sensitivity of 90.9%, specificity of 79.3%, and accuracy of 80.4%. Using the optimal CPS_norm_ calculated from all patients (CPS_norm_ = 79), combining the two modalities resulted in a lower sensitivity compared to that of the individual modalities, but increased the specificity to 87.4%.

## Discussion

This is the first trial to evaluate separately administered optical and radiolabeled monoclonal antibodies for preoperative imaging and intraoperative detection of lymph nodes in HNSCC. In 17 patients undergoing surgical resection, we found that [^111^In]panitumumab is safe, with no adverse events related to the study. We did see elongation in the QTc between pre- and post-infusion, however, it was not outside of clinically safe ranges and is a known side effect of panitumumab 44. Infusion of [^111^In]panitumumab greater than three days prior to SPECT/CT imaging could successfully detect primary and metastatic disease greater than 1 cm in size, similar to the findings be de Gooyer et al. in colorectal carcinoma 35. *In vivo*, the radiotracer localized to the primary tumor, however, lymph node detection demonstrated insufficient signal-to-background. Tumor specific accumulation of radiolabeled panitumumab within metastatic lymph nodes was better demonstrated *ex vivo* as evidenced by increased gamma counts compared to benign lymph nodes. These findings are consistent with prior clinical trials using intraoperative optical imaging, which demonstrates anti-EGFR antibodies can determine metastasis based on *ex vivo* signal intensity 15-17. Furthermore, we were able to demonstrate good co-localization of the dual antibodies, even when given in series as separate injections and not dual-labeled on the same antibody.

Our findings expand on the recent trials exploring the use of indium-111 dual-labeled monoclonal antibodies in renal and colorectal carcinoma, as their findings were primarily reported within the primary tumor or *ex vivo* use 34, 35. Our previous work has shown that fluorescently labeled panitumumab provides high SBR between readily identified lesions and the background 10, 40, 45, 46. Additionally, we have shown that pan800 can predict lymph node metastases with high specificity; however, clinical utility of *in vivo* use has been limited by low signal penetration depth 15, 17, 18. Using the same antibody, we substituted the optical label for a nuclear one with the expectation that the deeper signal penetration depth would allow for localization of metastatic tissue using a gamma probe which could then be confirmed with optical imaging. This model has been demonstrated abundantly in preclinical as well as recent clinical trials 27, 28, 34, 35, 47. In contrast to these studies, we obtained separate investigational approval for each molecular imaging agent and infused in sequence. Our results from separately injecting a gamma-emitting antibody probe and fluorescently labeled antibody probe show localization similar to those where the antibody was dual-labeled, suggesting the two approaches are equivalent. However, detection of the [^111^In]panitumumab *in vivo* was significantly limited by background activity as predicted by a previous paper simulating the use of indium-111 labeled antibodies by Martin and Thurston 33, which is not a problem in NIRF imaging. By showing the two tracers still accumulate in the tissue of interest when administered separately, we demonstrated the possibility of changing one modality to be more compatible with the other. This has practical implications for future trial development as it allows for independent optimization of dose and pharmacokinetics of each agent individually and in tandem to evaluate the best application for detection of cancer.

We chose indium-111 based on intraoperative validation from recent studies, half-life of around 2.8 days making it compatible with the monoclonal antibody, proven safety of the agent, and ability to detect emissions with commercially available gamma probes. Given the half-life of indium-111 and previously determined half-life of the antibody 13, we preformed imaging and surgery within 2-7 days. Unfortunately, we found that performing surgery towards the end of the interval improved the background counts but co-existed with decay of the indium-111 levels resulting in lack of meaningful counts. Previous trials in colorectal carcinoma using different radionuclides have been reported on extensively 19-22. The authors used iodine-131, a gamma-emitting compound with a half-life of 8 days, however, found radiation scattering and high background detection rendered the compound inadequate 33. Later trials used iodine-125 (half-life 59.4 days) where lesions *in vivo* were detected with sensitivities of 75-100% and positive predictive values of 69-75% 19, 48. The longer half-life of iodine-125 allowed the authors to achieve a high SBR, however, required infusing patients 20 or more days prior to surgery. Additionally, using iodine-based radionuclides such as iodine-131 or iodine-125 in patients with functioning thyroids necessitates giving potassium iodide to block thyroid uptake 33. The limited feasibility of the indium-111 labeled antibody for *in vivo* use is an important finding, especially when considering the ultimate goal of using the radiotracer to locate lesions not readily visible with fluorescence due to the shallow signal penetration. Aside from using a radionuclide with a half-life that exceeds the targeting antibody as in previous successful radio-immunoguided surgery (RIGS) trials, other methods to avoid high circulating background activity such as the use of beta-emitters or smaller molecules should be considered. We have previously reported results in patients with HNSCC who received pan800 and [^89^Zr]panitumumab showing the feasibility and high specificity of [^89^Zr]panitumumab for detecting lesions as small as lymph nodes on PET/CT imaging 37. While the half-life of zirconium-89 (3.27 days) is slightly longer than indium-111 (2.8 days), its emission of 511 keV is a higher energy than optimal for intraoperative detection 49. While using a PET agent like zirconium-89 could work in theory, very few commercially available beta-probes exist for intraoperative use 50.

There are several limitations to this study to consider. Given the trials were pilot studies with a sample size of 17, power is limited in assessing meaningful statistical differences. In contrast to previous dual-modality studies, we expanded the imaging and surgery window to better understand how the two modalities could be used together. Previous studies on pan800 have shown higher tumor MFI before day 3, and therefore, we determined the window based on these findings and prior experience 14. While NIRF was successful through day 7, the physical half-life of the radiotracer created variable gamma detection at differing time points thus limiting inter-patient comparisons as well as comparison between the two modalities for clinical use. Where our *ex vivo* findings were comparable to previous studies of pan800 alone in lymph nodes, the real goal was to improve *in vivo* utility. Directly comparing the *in vivo* results would not explain the full picture as they are meant to complement each other. Future studies to optimize the timing compatibility of the two modalities would be beneficial. Another limitation is with the surgical equipment as probe measurements are highly sensitive to distance and contact angle from target. All efforts were taken to ensure consistency between patients; however, future studies could limit variability by choosing patients with more favorable tumor locations and enrolling patients from 1-2 surgeons to maximize technique.

## Conclusion

Intravenous infusion of [¹¹¹In]panitumumab, alone or with pan800, is safe and feasible in patients with HNSCC. Antibody-based detection of metastatic lymph nodes using the radiotracer was limited *in vivo* by background activity. However, *ex vivo* analysis demonstrated reliable tumor-specific accumulation and strong correlation between optical and nuclear tracers. This study of antibody-based molecular imaging agents for intraoperative identification of metastatic lymph nodes highlights opportunities to improve sensitivity and specificity through tracer design and timing optimization.

## Figures and Tables

**Table 1:** Patient Characteristics

					Staging		Hours from Infusion	
ID	Primary Location	Age	Gender	Recurrence	Clinical	Pathologic	Imaging	Surgery
1	Oral Tongue	37	M	No	T3N2	T2N0	76	120
2	Base of Tongue	63	M	Yes	T4N0	yT4N0	74	118
3	Base of Tongue	58	M	Yes	T1N0	T1N0	27	94
4	Mandibular Gingiva	63	M	No	T2N0	T1N0	98	114
5	Oral Tongue	63	F	No	T2N0	T1N0	19	44
6	Maxillary Gingiva	49	M	No	T4aN1	T4aN1	117	137
7	Floor of Mouth	65	M	No	T3N0	T3N0	99	117
8	Mandibular Gingiva	69	M	No	T4aN1	T4aN0	71	93
9*	Oral Tongue	83	M	No	T1N0	T1N0	97	119
10*	Oral Tongue	53	F	No	T2N1	T3N1	97	116
11*	Base of Tongue	68	M	Yes	T3N1	rT4mN1	22	42
12*	Base of Tongue	60	M	No	T2N1	T2N2a	141	162
13*	Base of Tongue	63	M	Yes	T4aN1	rT4N0	118	140
14*	Mandibular Gingiva	55	F	No	T3N1	T4aN2b	92	112
15*	Mandibular Gingiva	81	F	No	T3N2c	T4aN3b	93	114
16*	Buccal Mucosa	76	M	No	T4aN1	T4aN3b	144	162
17*	Mandibular Gingiva	57	M	No	T4aN2b	T4aN0	122	138

*Patients enrolled in In-pan + pan800. M: Male; F: Female

## Abbreviations

AUC: area under the curve
CPS_norm_: normalized counts per second
CTCAE: Common Terminology Criteria for Adverse Events
EGFR: epidermal growth factor receptor
EKG: electrocardiogram
FDA: Food and Drug Administration
FGS: fluorescence guided surgery
cGMP: current Good Manufacturing Practice
HNSCC: head and neck squamous cell carcinoma
HPLC: high-performance liquid chromatography
IND: Investigational New Drug
IQR: interquartile range
LBM: lean body mass
MALDI-TOF MS: matrix-assisted laser desorption ionization time-of-flight mass spectrometry
MEGP: medium-energy general purpose
MFI: mean fluorescence intensity
NIRF: near-infrared fluorescence
pan800: panitumumab-IRDye800CW
RGS: radioguided surgery
ROC: receiver operating curve
ROI: region of interest
SBR: signal-to-background
SCN-Bn-DTPA: S-2-(4-Isothiocyanatobenzyl)-diethylenetriamine pentaacetic acid
SD: standard deviation
SLNB: sentinel lymph node biopsy
SPECT/CT: single photon emission computed tomography with computed tomography scan
TBP: tumor-to-blood pool ratio
VUIIS: Vanderbilt University Institute of Imaging Sciences
VUMC: Vanderbilt University Medical Center
WEHRS: wide-energy high-resolution sensitivity
